# Supplementary material for: MetaRibo-Seq measures translation in microbiomes
Source: Nat Commun. 2020 Jun 29;11:3268. doi: 10.1038/s41467-020-17081-z (PMC7324362; doi:10.1038/s41467-020-17081-z)
Supplement: Supplementary file 10 — Supplementary Data 7 [file 41467_2020_17081_MOESM10_ESM.zip › File2/Confidence_High_Taxonomy/352018_out.krona.html]

Javascript must be enabled to view this page.

members
magnitude
magnitudeUnassigned
count
unassigned
taxon
rank

352018\_out

3

superkingdom
3
2

976
phylum
3

class
3
200643

order
3
171549

3
family
171552

3
genus
1926672

species
3

SRS055017\_contig\_number\_19328SRS143070\_contig\_number\_3349SRS147271\_contig\_number\_10825
1852370
